# ^131^I therapy for benign thyroid disease: flexible single-time-point dosimetry using population-based model selection with non-linear mixed-effects modelling

**DOI:** 10.1186/s40658-025-00806-8

**Published:** 2025-10-13

**Authors:** Deni Hardiansyah, Ade Riana, Heribert Hänscheid, Jaja Muhamad Jabar, Ambros J. Beer, Michael Lassmann, Gerhard Glatting

**Affiliations:** 1 Medical Physics and Biophysics, Physics Department, Faculty of Mathematics and Natural Sciences, Universitas Indonesia, Depok, Indonesia; 2 Radiation Protection and Compliance Testing Laboratory, Medical Devices and Facilities Safety Center BPAFK, Jakarta, Indonesia; 3 Department of Nuclear Medicine, University Hospital Würzburg, Würzburg, Germany; 4 Nuclear Physics and Biophysics, Physics Department, Faculty of Mathematics and Natural Sciences, Institut Teknologi Bandung, Bandung, Indonesia; 5 Department of Nuclear Medicine, Ulm University, Ulm, Germany; 6 Medical Radiation Physics, Department of Nuclear Medicine, Ulm University, Ulm, Germany

**Keywords:** Single-time-point dosimetry, NLME modelling, ^131^I, Thyroid

## Abstract

**Purpose:**

This study aimed to evaluate the accuracy of time-integrated activity (TIA) estimation using single-time-point (STP) data combined with nonlinear mixed-effects modelling (NLMEM) and population-based model selection (PBMS) in 73 patients with benign thyroid disease.

**Methods:**

Biokinetic data of ^131^I were collected from 73 patients with benign thyroid conditions, including Graves’ disease, toxic nodular goitre, and non-toxic goitre. Uptake measurements were taken at 2, 6, 24, 48 (73 patients), 96 h (53 patients) or 120 h (20 patients) after administration. The best sum-of-exponentials function (SOEF) with four adjustable parameters, identified in our recent study (Hardiansyah et al. EJNMMI Phys, 2025) by PBMS NLMEM, and the SOEF EANM standard operating procedure (SOP) with three adjustable parameters were then employed to conduct a NLMEM-based STP dosimetry at different time points, i.e. s1TIA and s2TIAs, respectively. In addition, STP dosimetry was performed using the EANM SOP approach (Hänscheid et al. EJNMMI, 2013) to calculate TIAs (hTIAs). The accuracy of the computed s1TIAs, s2TIAs and hTIAs was evaluated by calculating the relative deviations (RDs), mean absolute percentage errors (MAPEs), root-mean-square errors (RMSEs), and percentage of absolute RDs of sTIAs and hTIAs exceeding 5% (%RD5) and 10% (%RD10) with the reference TIAs obtained from the four parameters NLMEM fit to all time points data.

**Results:**

Of the time points included, 120 h after administration was found as the optimal time point for STP dosimetry based on the mean ± SD of RD (RMSE, MAPE, %RD5, %RD10) of s1TIA, s2TIAs, and hTIA of 2% ± 4% (4%,3%,25%,0%), 13% ± 6% (14%,13%,90%,60%) and −3% ± 5% (6%,5%,55%,0%), respectively.

**Conclusions:**

While s1TIA typically produced better ^131^I TIA estimates for benign thyroid disease than the hTIA recommended in the EANM SOP, the STP calculation from NLMEM using SOEF EANM SOP s2TIAs was inferior to hTIA. With the best SOEF model, NLMEM provides satisfactory and reasonable STP estimates.

## Introduction

The primary objective of radioiodine therapy for benign thyroid disease is to treat toxic adenoma in euthyroidism, hyperthyroidism, or as part of the ablative concept in Graves’ disease, as well as to manage hypothyroidism compensated by thyroxine medication [1–3]. The goal of individual dosimetry before radioiodine therapy is to determine the appropriate activity of ^131^I to administer, ensuring a high absorbed dose to the target while limiting unnecessary radiation exposure [4]. Although there is increasing evidence that individual dosimetry improves the outcome and quality of radionuclide therapy, the requirement for multiple measurements of biokinetic data poses a challenge to its implementation in routine clinical practice [5, 6]. Multiple measurements for individual dosimetry in molecular radiotherapy necessitate several patient visits, resulting in a high workload and a significant burden [7, 8]. Therefore, an individual dosimetry method utilising single-time-point (STP) measurement of biokinetic data is highly desirable [7, 9].

The implementation of STP individual dosimetry in radionuclide therapy for calculating time-integrated activity (TIA) has been increasing over the years. Among the published STP methods, STP dosimetry using non-linear mixed effects modelling (NLMEM) is a favourable approach for [^177^Lu]Lu-DOTATATE [10, 11], [^177^Lu]Lu-PSMA-617 [6] and ^131^I [12] as it has been shown to have less bias than the frequently used STP method in the literature [6]. While STP dosimetry using NLMEM for ^131^I has demonstrated promising results, using a bi-exponential function for STP analysis, as applied in previous studies [12], may not be optimal and could introduce bias. Our recent study has emphasised that the selection of the fit function is a critical step in accurately estimating TIA for ^131^I dosimetry [13]. Population-based model selection (PBMS) combined with NLMEM has been demonstrated to outperform individual-based model selection [14] and PBMS with shared parameters [15]. We recently showed that using suboptimal fit functions, such as the EANM standard operating procedure (SOP) bi-exponential function [1], in ^131^I dosimetry introduces a bias of up to 29% [13]. Compared to the commonly used approach in defining the fit function for calculating the TIA (and subsequently the absorbed dose), the PBMS NLMEM method offers a more reproducible, objective, and standardised framework for establishing reference dosimetry in radionuclide therapy.

Furthermore, STP dosimetry with PBMS NLMEM has not yet been tested on diverse patient populations with thyroid disease. This testing is essential for assessing the accuracy of calculated TIA and improving dosimetry in ^131^I thyroid therapy. To address this gap, this study applied STP dosimetry, using the best fit function identified from the PBMS NLMEM for ^131^I [13], to a cohort of 73 patients with Graves’ disease, toxic nodular goitre, and non-toxic goitre undergoing ^131^I therapy.

## Methods

### Biokinetic data

Data on target tissue retention of ^131^I, the fraction of administered activity in the thyroid gland as a function of time, were collected from 73 patients: 12 with Graves’ disease, 59 with toxic nodular goitre, and 2 with non-toxic goitre. Measurements were taken at 2, 6, 24, 48 h (n = 73), and 96 h (n = 53) or 120 h (n = 20) after oral administration of 1 MBq ^131^I. One patient did not have a 6-h measurement, resulting in 364 data points for the analysis. The measurement process utilised a collimated 2 × 2” NaI crystal equipped with a single-channel analyser, which underwent regular quality checks, daily for background levels and sensitivity and weekly for the energy spectrum. Biokinetic data were derived by calculating the difference in count rates detected over the neck at 35 cm and over the thigh simultaneously to account for blood pool activity. This difference was then standardised against the count rate obtained from the ^131^I capsule placed within a thyroid phantom before administration.

### Fit function: sums of exponential functions

The sum-of-exponentials function (SOEF) ${a}_{4c}\left(t\right)$ that has been shown as the best model to describe the biokinetics of ^131^I in our patient population [13] based on PBMS NLMEM and, as an example of a less well-fitting function, the EANM SOP function ${a}_{3b}\left(t\right)$ were used in this study:

1 $${a}_{4c}\left(t\right)=\frac{{\lambda }_{1}}{{\lambda }_{2}+{\lambda }_{1}-{\lambda }_{3}}\left({e}^{-\left({\lambda }_{3}+{\lambda }_{phys}\right)t}- {e}^{-\left({\lambda }_{1}+{\lambda }_{2}+{\lambda }_{phys}\right)t}\right)+{a}_{1}{e}^{-\left({\lambda }_{1}+{\lambda }_{2}+{\lambda }_{phys}\right)t}$$

2 $${a}_{3b}\left(t\right)=\frac{{\lambda }_{1}}{{\lambda }_{2}+{\lambda }_{1}-{\lambda }_{3}}\left({e}^{-\left({\lambda }_{3}+{\lambda }_{phys}\right)t}- {e}^{-\left({\lambda }_{1}+{\lambda }_{2}+{\lambda }_{phys}\right)t}\right)$$

where ${a}_{px}(t)$ is a SOEF with $p$ estimated parameters and index $x$ used in [13] for identification, the ${a}_{1}$ is the initial amplitude of the respective exponential term, ${\lambda }_{phys}$ is the physical decay constant of ^131^I (${\lambda }_{phys}=\text{ln}\left(2\right)/{T}_{1/2}$, with the ^131^I half-life ${T}_{1/2}$=8.022 d [16]), ${\lambda }_{index}$ are the biological clearance or uptake rates of the radiopharmaceutical with values $\ge 0$ [17, 18]. SOEF ${a}_{4c}\left(t\right)$ is the EANM SOP equation [16] with the additional term ${a}_{1}\cdot {e}^{-({\lambda }_{2}+{\lambda }_{1}+{\lambda }_{p})\cdot t}$. This term represents the contribution of the blood pool with factor ${a}_{1}$ which takes into account any residual count rate from tissue activity after subtraction of the thigh measurement [13].

### Study workflow

**Fig. 1:**
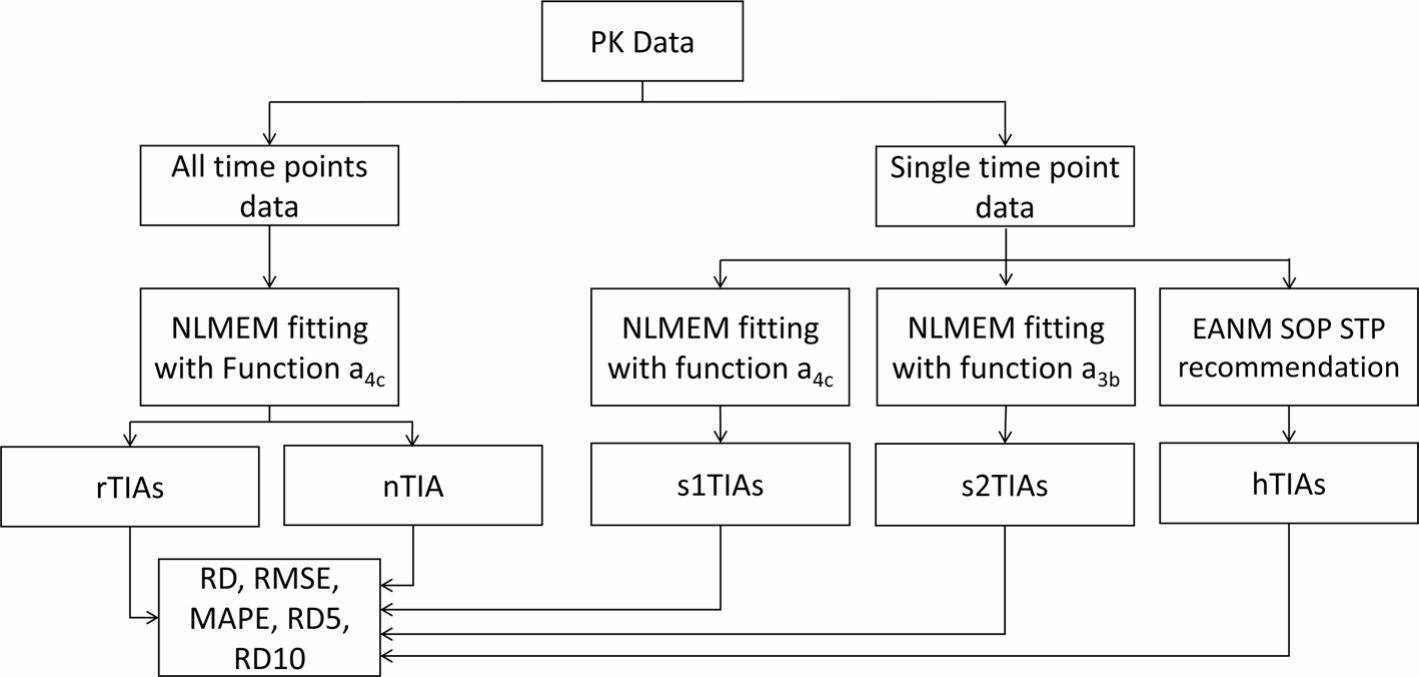
Workflow of the study. The SOEF a_4c_, identified as the best function for describing ^131^I biokinetic data from the PBMS NLMEM approach [13], was used. The reference TIAs (rTIAs) were calculated based on all-time-point NLMEM fittings using SOEF a_4c_. Single-time-point TIAs (s1TIAs and s2TIAs) were computed using only STP data and NLMEM for each patient. Additionally, single-time-point TIAs (hTIAs) were calculated using Eq. (3) (EANM SOP [1]). The RDs, RMSEs, MAPE, RD5 and RD10 were employed to analyse the accuracy of the s1TIAs, s2TIAs, and hTIAs, with rTIAs as the reference values. The nTIA was analytically calculated by determining the mean value of the parameters in Eq. (1) of all patients from the NLMEM all-time-point fit to mimic the dosimetry without any patient measurement, i.e. the no-time-point (NTP) case

The workflow of the study is shown in Fig. 1. The parameters of Eq. (1) were fitted to the all-time-point biokinetic data of ^131^I (Sect. 2.1) within the NLMEM framework [19–21] using function ${a}_{4c}\left(t\right)$ to derive the reference TIA (rTIA). The NLMEM simulations and fittings were performed using MATLAB software version R2020a (https://www.mathworks.com/help/stats/nlmefitsa.html). A thousand random starting values were generated to find the best starting value for each NLMEM fitting. The TIA was calculated analytically from function ${a}_{4c}\left(t\right)$.

In STP dosimetry, the SOEF parameters of function ${a}_{4c}\left(t\right)$ and the EANM SOP function ${a}_{3b}\left(t\right)$ were fitted using a NLMEM for each patient’s STP biokinetic data at different time points, incorporating biokinetic data from all other patients. The Jackknife (leave-one-out cross-validation) method was applied to each STP fitting to assess and validate the robustness of the method. STP TIAs were analytically calculated using the fitted parameters of function ${a}_{4c}\left(t\right)$ (s1TIAs) and the EANM SOP function ${a}_{3b}\left(t\right)$ (s2TIAs).

For comparison, the recommendations of the EANM SOP [1, 16] to calculate the TIAs from individual retention measurements (hTIAs) were implemented in this study using the following functions:

3a $$hTI{A}_{i}=0.97\times A\left({t}_{i}\right)\times \frac{{2}^{\frac{{t}_{i}}{5.5 d}}\times 5.5 d}{\text{ln}(2)}, \forall 1\text{ d }\le {t}_{i} \le 3\text{ d}$$

3b $$hTI{A}_{i}=A\left({t}_{i}\right)\times \frac{{t}_{i}}{0.357}, \forall 4\text{ d }\le {t}_{i} \le 8\text{ d}$$

where $A\left({t}_{i}\right)$ is the activity measured at time point $i$ and ${t}_{i}$ is the time of measurement $i$ [1, 16]. According to the EANM SOP, the calculation of hTIA is recommended for late time point measurements [1]. Therefore, in this study, hTIA was calculated only for biokinetic measurements at 24, 48, 96, and 120 h after administration. In addition, no-time-point TIA (nTIA) was calculated analytically using the mean of the parameters in Eq. (1) derived from the NLMEM fitting to surrogate the dosimetry with no-time-point (NTP) imaging measurement.

Analyses of the accuracy of the STP TIAs were done based on the relative deviation (RD), root-mean-square errors (RMSE), and mean absolute percentage error (MAPE) with the rTIA as the reference:

4 $${RD}_{k,j}=\frac{{sTIA}_{k,j}-{rTIA}_{j}}{{rTIA}_{j}},$$

5 $${RMSE}_{k}=\sqrt{{\left({SDRD}_{k}\right)}^{2}+{\left({MeanRD}_{k}\right)}^{2}}$$

6 $${MAPE}_{k}=\text{mean}\left|\frac{{sTIA}_{k,j}-{rTIA}_{j}}{{rTIA}_{j}}\right|,$$

where ${RD}_{k,j}$ is the relative deviation of the STP method (s1TIA, s2TIA, hTIA, or nTIA) at time point $k$ and patient$j$, ${RMSE}_{k}$ is the root-mean square over all patients of ${RD}_{k}$ at time point$k$, ${SDRD}_{k}$ is the standard deviation of ${RD}_{k,j}$ in all patients at time point $k$, ${MeanRD}_{k}$ is the mean of ${RD}_{k,j}$ in all patients at time point$k$, and ${MAPE}_{k}$ is the mean absolute percentage error in all patients at time point $k$. The total number of patients with absolute RDs of sTIAs, hTIAs, and nTIA higher than 5% (RD5), 10% (RD10), and 20% (RD20) was also calculated and analysed.

The Wilcoxon matched-pairs signed-rank test with *p* < 0.05 (using GraphPad Prism version 10.6.0 for Windows, GraphPad Software, Boston, Massachusetts, USA, www.graphpad.com) was performed to determine if the difference between TIACs from STP dosimetry using NLMEM and the EANM SOP recommendation is statistically significant.

## Results

**Fig. 2:**
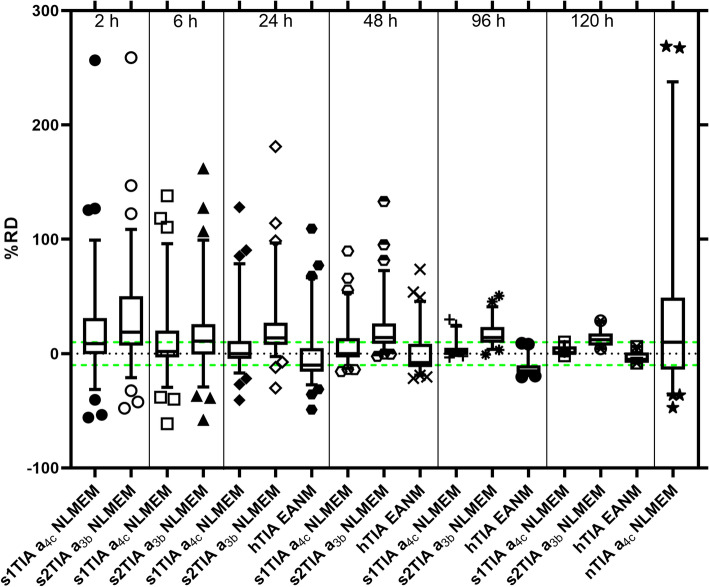
Relative deviation (RD) values of s1TIAs derived from fitting the STP data with SOEF a_4c_, s2TIAs derived from fitting the STP data with SOEF a_3b_ within the NLMEM framework, hTIAs obtained from Eq. (3) published in the EANM SOP [1], and nTIA a_4c_ NLMEM. The TIAs from all-time-point NLMEM fitting (rTIAs) were used as the reference

Figure 2 shows the RDs of the s1TIA, s2TIAs, and hTIA at different time points. The (mean ± standard deviation) of RD (RMSE, MAPE, RD5, and RD10) of hTIA and sTIA from both functions ${a}_{3b}\left(t\right)$ and ${a}_{4c}\left(t\right)$ are shown in Table 1. The mean RD (RMSE, MAPE) values of s1TIA from function ${a}_{4c}\left(t\right)$ were lower in all investigated STP time points. Both the s1TIA from function ${a}_{4c}\left(t\right)$ and the EANM recommendation hTIA outperformed the s2TIA from ${a}_{3b}\left(t\right)$ in predicting the rTIA (Table 1) based on the RD (RMSE, MAPE, RD5, and RD10) values. In general, a measurement taken 120 h after administration was the best time point for STP dosimetry for s1TIAs, s2TIAs, and hTIAs in our dataset.

**Table 1:** Performance of the STP dosimetry with the PBMS NLMEM and EANM SOP methods

Method	Used single time point	%RD mean ± SD	%RMSE	%MAPE	Patients with RD > 5% (%)	Patients with RD > 10% (%)	Patients with RD > 20% (%)
SOEF ${a}_{4c}\left(t\right)$	2 h	19 ± 44	48	28	58 (79)	47 (64)	31 (42)
	6 h	12 ± 34	36	21	46 (63)	36 (49)	26 (36)
	24 h	8 ± 27	28	16	41 (56)	31 (42)	18 (25)
	48 h	7 ± 19	21	12	38 (52)	24 (33)	15 (21)
	96 h*	4 ± 8	9	4	13 (25)	8 (15)	4 (8)
	120 h**	**2 ± 4**	**5**	**3**	**5 (25)**	**1 (5)**	**0 (0)**
SOEF ${a}_{3b}\left(t\right)$	2 h	31 ± 45	55	36	67 (92)	59 (81)	39 (53)
	6 h	17 ± 35	39	25	61 (84)	48 (66)	28 (39)
	24 h	23 ± 30	38	24	64 (88)	51 (70)	29 (40)
	48 h	22 ± 24	33	22	65 (89)	50 (68)	27 (37)
	96 h*	17 ± 11	20	17	49 (92)	35 (66)	18 (34)
	120 h**	**13** **± 6**	**14**	**13**	**18 (90)**	**12 (60)**	**2 (10)**
EANM SOP (Eqs. (3a and 3b))	24 h	− 2 ± 26	26	19	61 (84)	52 (71)	21 (29)
	48 h	0 ± 19	19	14	60 (82)	42 (58)	15 (21)
	96 h*	− 12 ± 7	14	14	50 (94)	40 (75)	2 (4)
	120 h**	**− 3 ± 5**	**6**	**5**	**11 (55)**	**0 (0)**	**0 (0)**
NTP	–	35 ± 76	83	50	63 (86)	56 (77)	43 (59)

*The total number of patients is 53

**The total number of patients is 20; Bold values indicate the time points resulting in the lowest MAPE for each STP dosimetry method.

The two-tailed Wilcoxon matched-pairs signed rank test demonstrated statistically significant differences (all *p* values < 0.0001) in RDs for STP dosimetry at 24 h, 48 h, 96 h, and 120 h after oral administration between the EANM SOP STP recommendation and NLMEM with function ${a}_{4c}\left(t\right)$ with median of differences of 9.9%, 6.7%, 16.2%, and 5.9%, respectively. The size of the effect, i.e., the accuracy of the RDs in both STP methods, was comparable. However, the percentage of cases with a deviation of more than 5% was generally lower with the NLMEM method. The NTP dosimetry showed the lowest performance compared to other methods (Table 1).

**Fig. 3:**
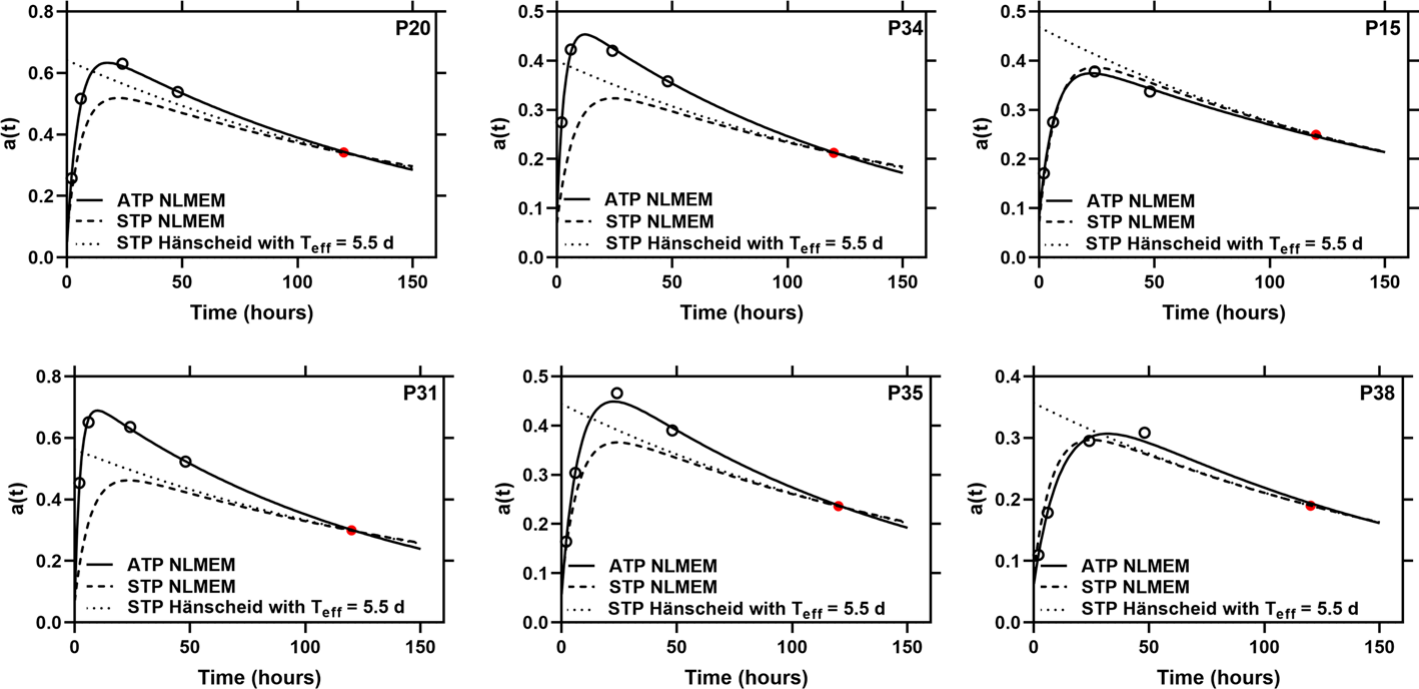
Time-activity curves from all-time-points (solid lines) and STP fittings (dotted lines) at time point 120 h post administration simulated with SOEF a_4c_ for 4 patients with the highest RDs (P20 with RD = 7.5%, P31 with RD = 8.7%, P34 with RD = 7.2% and P35 with RD = 9.8%) and patients with RDs at the 25th (P15 with RD = − 1.2%) and 75th percentiles (P38 with RD = 5.9%) in the population

Four patients out of a total of 73 patients had an RD outside the (mean ± 2SD) of the population RD (Fig. 3) from the STP dosimetry with function ${a}_{4c}\left(t\right)$ at 120 h after administration. In all these patients, the s1TIAs values were higher than the rTIAs. In addition to these patients, Fig. 3 displays the fitted curves for typical patients P15 and P38, which correspond to the 25th and 75th percentiles in the population.

## Discussion

This study investigated the accuracy of STP dosimetry using NLMEM in ^131^I therapy. The SOEF function ${a}_{4c}$ identified as the best function for 364 biokinetic data of ^131^I from the PBMS NLMEM method [13] was used for defining the reference in this study. As a result, the STP biokinetic data of ^131^I measured 120 h after administration was found to have the lowest mean ± standard deviation of RD (RMSE, MAPE, %RD5, %RD10) of 2% ± 4% (5%, 3%, 25%, 5%) for the calculation of s1TIA using NLMEM from all investigated uptake measurement time points (Sect. 2.1). The STP dosimetry method from the EANM SOP [1] for calculating the hTIA performed similarly at 120 h after administration, with a mean ± standard deviation of RD (RMSE, MAPE, %RD5, %RD10) of −3% ± 5% (6%, 5%, 55%, 0%). This result indicates that the PBMS NLMEM method outperforms STP dosimetry according to the EANM SOP [1] regarding the probability for deviations exceeding 5% (%RD5).

Our findings demonstrate that the accuracy of STP dosimetry improved when measurements were taken at later time points, which can be explained by the fact that measurements after 4–8 days, preferably 6–7 days, are most representative of the TIA (supplement of [1]). Specifically, the optimal method for determining TIA in the thyroid was the combination of PBMS NLMEM and STP imaging of ^131^I kinetics at 120 h after administration. This approach reduces the likelihood of significant deviations, as evidenced by only four “outliers” in our data (from a total of 73 patients), defined as RDs falling outside the mean ± 2SD range (Table 1). However, all RDs of s1TIAs at 120 h were below 10% as shown by the RD10 value in Table 1. These results highlight the potential of late-time-point measurements to enhance the accuracy of thyroid dosimetry, thereby supporting their incorporation into clinical workflows.

Nevertheless, in scenarios where late-time-point measurements are impractical, STP dosimetry conducted at earlier time points, e.g. at 24 or 48 h after administration, may still provide acceptable accuracy, depending on clinical considerations (Table 1). Additionally, selecting an appropriate fit function is crucial for achieving reliable STP dosimetry accuracy using NLMEM. Our analysis demonstrated that the STP method employing NLMEM with function ${a}_{4c}\left(t\right)$ outperformed the STP method using ${a}_{3b}\left(t\right)$ at 120 h after administration, as shown in Table 1 and Fig. 2. This observation aligns with our recent study [13], which underscored the importance of model selection and highlighted the role of parameter a_1_ in function ${a}_{4d}\left(t\right)$. These findings emphasise the importance of optimising both time-point selection and SOEF choice to achieve accurate STP dosimetry results. The results also highlight the need for careful adaptation of NLMEM functions and parameters when applying them to similar applications. The parameter a_1_ in equation a_4c_(t), for example, depends on the measuring device and geometry as well as on the body site of the blood pool background measurement. Assuming an imprecise model structure in STP dosimetry may introduce systematic deviations in the estimated time course of the activity function, potentially leading to over- or underestimations of the TIAs. To minimise such risks, a PBMS from a well-thought-out model set is necessary when applying NLMEM for STP dosimetry.

The results of this study also align with previous research that has identified late time points as optimal for STP dosimetry using ^131^I. Specifically, STP data at 120 h after administration have been demonstrated to be the optimal time point for ^131^I dosimetry according to the SOP EANM function, as noted by Hänscheid et al. [16]. Other findings from dosimetry studies by Bockisch et al. [22], Merril et al. [12], and Joensson et al. [23] further support this conclusion. The studies reported that late uptake measurements taken around 192 h [22], 168 h [12], and 96 h [23] showed a reasonable accuracy of TIA in the thyroid, with deviations of less than 5%. Therefore, our results, which highlight the importance of the 120-h time point after administration, are consistent with existing literature, showing the reliability of late-time measurements in accurately assessing thyroid dosimetry with ^131^I.

Although our findings indicate that STP dosimetry for ^131^I achieves optimal accuracy when performed at 120 h post-administration, a recent study by Gomes et al. [24] suggested that STP dosimetry remains clinically acceptable for [^177^Lu]Lu-PSMA-I&T even with a MAPE below 27%. If we assume the same threshold for our data, STP dosimetry for ^131^I could potentially be performed as early as 2 h post-administration, where we observed a MAPE of 28%. This suggests that a more flexible imaging schedule may be feasible, which could enhance the practicality and patient compliance of STP dosimetry in routine clinical settings. However, as shown in Fig. 2, for some patients this may lead to extremely high RD values up to 257%.

The < 27% MAPE threshold previously reported for [^1^⁷⁷Lu]Lu-PSMA-I&T, however, cannot be directly applied to benign thyroid disease, as treatment aims and clinical decision-making criteria differ substantially between the two settings. Nonetheless, it may provide a useful reference point when interpreting prediction accuracy, while recognising that for radioiodine therapy, a different, clinically relevant MAPE threshold may be more appropriate. Establishing such a threshold would require dedicated analysis of the dose–effect relationship in benign thyroid disease, taking into account that physicians also integrate additional covariates, such as patient characteristics and disease-specific factors, into activity prescription.

In this study, we applied STP dosimetry to the same patient population used for model selection, as initial validation on the same dataset is essential to establish baseline accuracy. Subsequent testing on independent datasets, particularly in multicentre settings, is required to assess potential bias and ensure generalizability. While the structural model is expected to remain consistent for the same radiopharmaceutical, parameter distributions may vary, underscoring the value of deriving models from large, diverse populations, as in our cohort of 73 ^131^I patients. The resulting population parameters can serve as prior knowledge for implementing STP dosimetry in other centres.

The structural model for ^131^I PBMS NLMEM was derived from the entire patient cohort to increase the ratio of observations to parameters, improving model characterisation and stability. Given the small and imbalanced disease subgroups, disease-specific model selection would have been less robust and potentially biased STP dosimetry. While disease-specific models were not assessed in this study, future studies in larger, balanced cohorts, potentially incorporating covariate selection, could provide further insight, including whether such an approach reduces outliers.

Although STP NLMEM dosimetry in our study demonstrated only a modest improvement over the STP EANM methodology at its optimal time point, 120 h post oral administration, the EANM approach is constrained to a narrow range of measurement times. In contrast, STP NLMEM maintained consistently high accuracy across all investigated time points (Table 1), offering greater flexibility for STP dosimetry implementation, particularly when acquisition times deviate from guideline recommendations. In addition, the NLMEM framework allows the incorporation of relevant covariates, such as patient-specific or disease-related factors, which can further improve predictive performance and enable more individualised dosimetry. While STP NLMEM is technically more complex, integration into user-friendly software can fully automate the process, enabling rapid, seamless clinical application without adding to the user’s workload.

Despite the promising results of this study, several limitations must be considered:

1. This study utilised biokinetic data of ^131^I in 73 patients with thyroid disease. While a larger dataset could provide a more refined and robust SOEF for STP dosimetry, our dataset is among the largest populations for ^131^I thyroid therapy reported in the literature. The results demonstrate that STP dosimetry performs with relatively good accuracy in this cohort. Nevertheless, validation with a larger and more diverse population would be beneficial to further generalise these findings.
2. The variability in fixed and random effects across different thyroid diseases in NLMEM, including Graves’ disease, toxic nodular goitre, and non-toxic goitre, represents a potential limitation. However, analysing all thyroid diseases collectively in this study allowed for a comprehensive assessment of STP dosimetry performance across a heterogeneous patient cohort, supporting its applicability across different thyroid disease types.
3. The modelling approach may not be fully optimised for the non-toxic goitre subgroup, as only two patients with this condition were included. This small sample size limits the ability to draw definitive conclusions about the accuracy of STP dosimetry in this population. Future studies with a larger non-toxic goitre cohort are necessary to validate and refine the dosimetry approach for this specific subgroup.
4. This study did not incorporate covariate analysis, which could have improved the predictive accuracy of STP dosimetry within NLMEM. Including patient-specific factors such as age, sex, and thyroid volume might enhance the model’s ability to describe inter-individual variability in the population. Future research should explore the integration of covariates to optimise STP dosimetry performance further.

## Conclusions

In this study, we developed and implemented an innovative STP dosimetry approach using PBMS NLMEM to calculate TIAs of ^131^I in a heterogeneous cohort of 73 patients. Our findings demonstrate that a single measurement of ^131^I biokinetics at 120 h after administration is optimal for accurately estimating thyroid TIAs. Compared to conventional multi-time-point dosimetry, this method simplifies the dosimetric workflow by reducing the number of required measurements and outperforms the STP approach recommended by the EANM SOP as reported in the literature [16]. Our findings suggest that the PBMS NLMEM approach represents a promising alternative to the EANM SOP STP recommendation for thyroid radioiodine therapy. Although its implementation is inherently more complex, it may support improved clinical outcomes by enabling more accurate and individualised dosimetry.

## Data Availability

The used data are available from the corresponding author upon reasonable request.

## Abbreviations

hTIAs: Time-integrated activity derived from single-time-point dosimetry using EANM SOP
MAPE: Mean absolute percentage error
NLMEM: Non-linear mixed-effects modelling
NTP: No-time-point
nTIA: No-time-point TIA
PBMS: Population-based model selection
RD: Relative deviation
RD5: Total number of patients with absolute RDs > 5% for specific STP methods
RD10: Total number of patients with absolute RDs > 10% for specific STP methods
RD20: Total number of patients with absolute RDs > 20% for specific STP methods
RMSE: Root-mean-square errors
rTIA: Reference TIA
s1TIA: Time-integrated activity derived from single-time-point dosimetry using NLMEM function a_4c_(t)
s2TIA: Time-integrated activity derived from single-time-point dosimetry using NLMEM function a_3b_(t)
sTIA: Time-integrated activity derived from single-time-point dosimetry using NLMEM
SOEF: Sum-of-exponentials function
SOP: Standard operating procedure
STP: Single-time-point
TIA: Time-integrated activity

## References

[1] Hänscheid H, et al. EANM dosimetry committee series on standard operational procedures for pre-therapeutic dosimetry II. Dosimetry prior to radioiodine therapy of benign thyroid diseases. Eur J Nucl Med Mol Imaging. 2013;40(7):1126–34. 23576099 10.1007/s00259-013-2387-x

[2] Di Martino F, et al. A theoretical model for prescription of the patient-specific therapeutic activity for radioiodine therapy of Graves’ disease. Phys Med Biol. 2002;47(9):1493–9. 12043815 10.1088/0031-9155/47/9/305

[3] Campenni A, et al. The EANM guideline on radioiodine therapy of benign thyroid disease. Eur J Nucl Med Mol Imaging. 2023;50(11):3324–48. 37395802 10.1007/s00259-023-06274-5 PMC10542302

[4] Willegaignon J, et al. Determining thyroid ^131^I effective half-life for the treatment planning of Graves’ disease. Med Phys. 2013;40(2):022502. 23387769 10.1118/1.4788660

[5] Hänscheid H, et al. Dose mapping after endoradiotherapy with ^177^Lu-DOTATATE/DOTATOC by a single measurement after 4 days. J Nucl Med. 2018;59(1):75–81. 28588150 10.2967/jnumed.117.193706

[6] Hardiansyah D, et al. Single-time-point renal dosimetry using nonlinear mixed-effects modeling and population-based model selection in [^177^Lu]Lu-PSMA-617 therapy. J Nucl Med. 2024;65(4):566–72. 38423787 10.2967/jnumed.123.266268

[7] Vergnaud L, et al. A review of ^177^Lu dosimetry workflows: how to reduce the imaging workloads? EJNMMI Phys. 2024;11(1):65. 39023648 10.1186/s40658-024-00658-8 PMC11554969

[8] Zhao W, et al. Accuracy of kidney dosimetry performed using simplified time activity curve modelling methods: a (177)Lu-DOTATATE patient study. Phys Med Biol. 2019;64(17):175006. 31287093 10.1088/1361-6560/ab3039

[9] Gustafsson J, Taprogge J. Theoretical aspects on the use of single-time-point dosimetry for radionuclide therapy. Phys Med Biol. 2022;67(2):025003. 10.1088/1361-6560/ac46e0 34965519

[10] Devasia T, et al. A novel time-activity information sharing approach using nonlinear mixed models for patient-specific dosimetry with reduced imaging time points: application in SPECT/CT imaging post ^177^Lu-DOTATATE. J Nucl Med. 2020;62(8):1118–25. 33443063 10.2967/jnumed.120.256255 PMC8833869

[11] Hardiansyah D, et al. Single-time-point dosimetry using model selection and nonlinear mixed-effects modelling: a proof of concept. EJNMMI Phys. 2023;10(1):12. 36759362 10.1186/s40658-023-00530-1 PMC9911583

[12] Merrill S, et al. Accuracy and optimal timing of activity measurements in estimating the absorbed dose of radioiodine in the treatment of Graves’ disease. Phys Med Biol. 2011;56(3):557–71. 21212469 10.1088/0031-9155/56/3/003

[13] Hardiansyah D, et al. Non-linear mixed-effects modelling and population-based model selection for 131I kinetics in benign thyroid disease. EJNMMI Phys. 2025. 10.1186/s40658-025-00735-6. 40198532 10.1186/s40658-025-00735-6 PMC11979076

[14] Kletting P, Glatting G. Model selection for time-activity curves: the corrected Akaike information criterion and the F-test. Z Med Phys. 2009;19(3):200–6. 19761098 10.1016/j.zemedi.2009.05.003

[15] Hardiansyah D, et al. A population-based method to determine the time-integrated activity in molecular radiotherapy. EJNMMI Phys. 2021;8(1):82. 34905131 10.1186/s40658-021-00427-x PMC8671591

[16] Hänscheid H, Lassmann M, Reiners C. Dosimetry prior to I-131-therapy of benign thyroid disease. Z Med Phys. 2011;21(4):250–7. 21531122 10.1016/j.zemedi.2011.01.006

[17] Williams LE, et al. On the correction for radioactive decay in pharmacokinetic modeling. Med Phys. 1995;22(10):1619–26. 8551986 10.1118/1.597421

[18] Glatting G, Reske SN. Treatment of radioactive decay in pharmacokinetic modeling: influence on parameter estimation in cardiac ^13^N-PET. Med Phys. 1999;26(4):616–21. 10227364 10.1118/1.598561

[19] Mould DR, Upton RN. Basic concepts in population modeling, simulation, and model-based drug development-part 2: introduction to pharmacokinetic modeling methods. CPT Pharmacometrics Syst Pharmacol. 2013;2:e38. 23887688 10.1038/psp.2013.14 PMC3636497

[20] Owen JS, Fiedler-Kelly J. Introduction to population pharmacokinetic/pharmacodynamic analysis with nonlinear mixed effects models. Hoboken: Wiley; 2014. 10.1038/psp.2014.51 PMC5684957 25531560

[21] Bonate PL. Pharmacokinetic-pharmacodynamic modeling and simulation. New York: Springer; 2011.

[22] Bockisch A, et al. Optimized dose planning of radioiodine therapy of benign thyroidal diseases. J Nucl Med. 1993;34(10):1632–8. 8410273

[23] Jonsson H, Mattsson S. Single uptake measurement for absorbed dose planning for radioiodine treatment of hyperthyroidism. Cancer Biother Radiopharm. 2003;18(3):473–9. 12954135 10.1089/108497803322285233

[24] Gomes CV, et al. Characterization of effective half-life for instant single-time-point dosimetry using machine learning. J Nucl Med. 2025;66(5):778–84. 40113223 10.2967/jnumed.124.268175 PMC12051766

