# Correction: ^131^I therapy for benign thyroid disease: flexible single-time-point dosimetry using population-based model selection with non-linear mixed-effects modelling

**DOI:** 10.1186/s40658-025-00818-4

**Published:** 2025-12-11

**Authors:** Deni Hardiansyah, Ade Riana, Heribert Hänscheid, Jaja Muhamad Jabar, Ambros J. Beer, Michael Lassmann, Gerhard Glatting

**Affiliations:** 1 Medical Physics and Biophysics, Physics Department, Faculty of Mathematics and Natural Sciences, Universitas Indonesia, Depok, Indonesia; 2 Radiation Protection and Compliance Testing Laboratory, Medical Devices and Facilities Safety Center BPAFK, Jakarta, Indonesia; 3 Department of Nuclear Medicine, University Hospital Würzburg, Würzburg, Germany; 4 Nuclear Physics and Biophysics, Physics Department, Faculty of Mathematics and Natural Sciences, Institut Teknologi Bandung, Bandung, Indonesia; 5 Department of Nuclear Medicine, Ulm University, Ulm, Germany; 6 Medical Radiation Physics, Department of Nuclear Medicine, Ulm University, Ulm, Germany

**Correction to: EJNMMI Physics (2025) 12:89** 10.1186/s40658-025-00806-8

Following publication of the original article [1], the figures and the corresponding captions were found swapped. The correct version should be as follows:

**Fig. 1:**
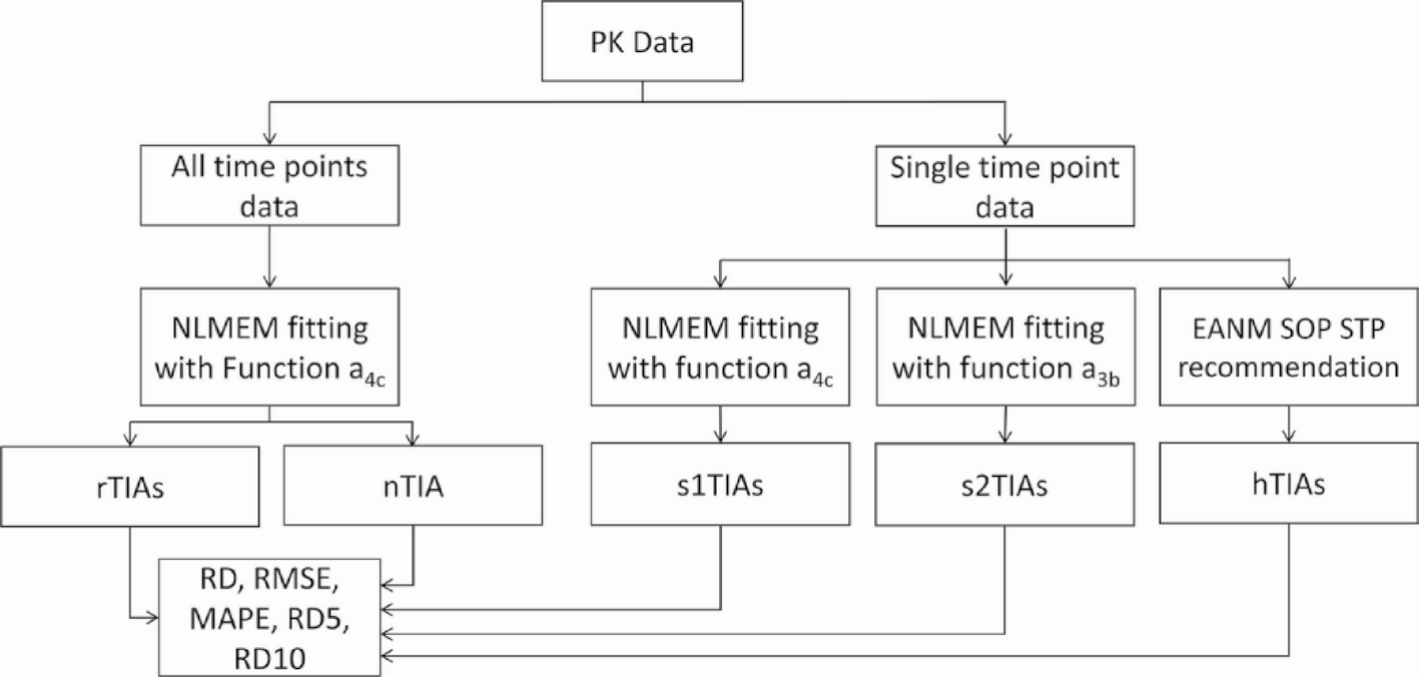
Workflow of the study. The SOEF a_4c_, identified as the best function for describing ^131^I biokinetic data from the PBMS NLMEM approach [13], was used. The reference TIAs (rTIAs) were calculated based on all-time-point NLMEM fittings using SOEF a_4c_. Single-time-point TIAs (s1TIAs and s2TIAs) were computed using only STP data and NLMEM for each patient. Additionally, single-time-point TIAs (hTIAs) were calculated using Eq. (3) (EANM SOP [1]). The RDs, RMSEs, MAPE, RD5 and RD10 were employed to analyse the accuracy of the s1TIAs, s2TIAs, and hTIAs, with rTIAs as the reference values. The nTIA was analytically calculated by determining the mean value of the parameters in Eq. (1) of all patients from the NLMEM all-time-point fit to mimic the dosimetry without any patient measurement, i.e. the no-time-point (NTP) case

**Fig. 2:**
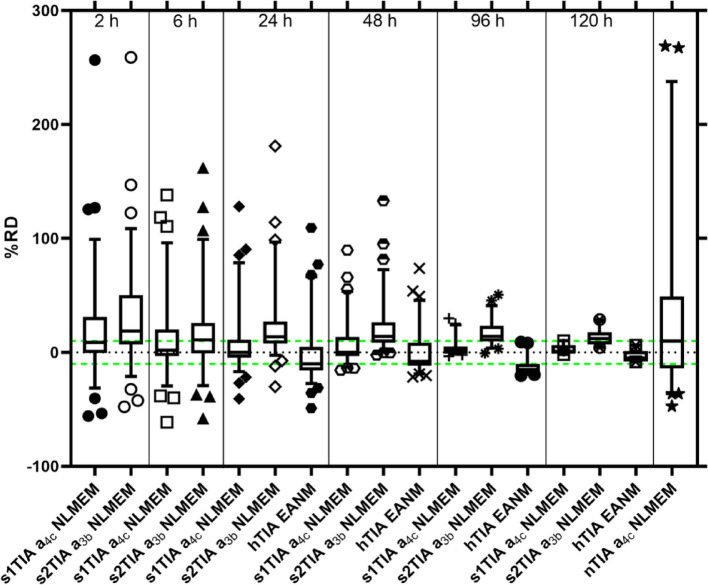
Relative deviation (RD) values of s1TIAs derived from fitting the STP data with SOEF a_4c_, s2TIAs derived from fitting the STP data with SOEF a_3b_ within the NLMEM framework, hTIAs obtained from Eq. (3) published in the EANM SOP [1], and nTIA a_4c_ NLMEM. The TIAs from all-time-point NLMEM fitting (rTIAs) were used as the reference

**Fig. 3:**
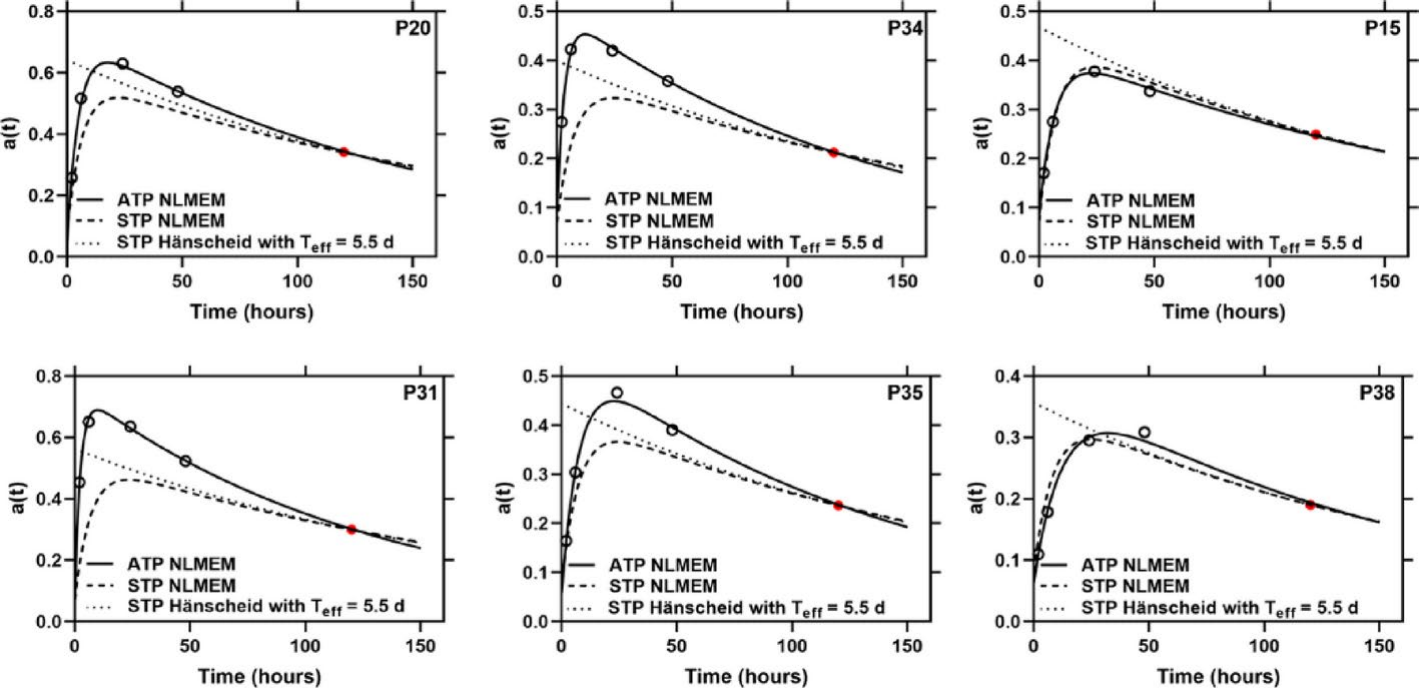
Time-activity curves from all-time-points (solid lines) and STP fittings (dotted lines) at time point 120 h post administration simulated with SOEF a_4c_ for 4 patients with the highest RDs (P20 with RD = 7.5%, P31 with RD = 8.7%, P34 with RD = 7.2% and P35 with RD = 9.8%) and patients with RDs at the 25th (P15 with RD = − 1.2%) and 75th percentiles (P38 with RD = 5.9%) in the population

The original article [1] has been updated.
